# Perspective: Making Treatment Decisions for Crohn’s Disease in 2025—Key Considerations

**DOI:** 10.3390/jcm14196879

**Published:** 2025-09-28

**Authors:** Mario Andrea Latorre, Michael Rennie, Konstantina Rosiou, Christian Philipp Selinger

**Affiliations:** 1 Leeds Gastroenterology Institute, Leeds Teaching Hospitals NHS Trust, Leeds LS9 7TF, UK; marioandrea.latorre01@universitadipavia.it (M.A.L.); michael.rennie2@nhs.net (M.R.); konstantina.rosiou@nhs.net (K.R.); 2 Department of Internal Medicine and Medical Therapeutics, University of Pavia, 27100 Pavia, Italy

**Keywords:** inflammatory bowel disease, Crohn’s disease, improved care

## Abstract

The treatment algorithm for Crohn’s disease evolves over time. In this review article we highlight nine studies from the last decade that have influenced our thinking on the treatment of Crohn’s disease. This perspective is a narrative, opinion-based review and reflects our personal thinking on some aspects of the treatment of Crohn’s disease. We selected original studies that have influenced our thoughts on luminal Crohn’s disease treatment significantly. Moderate to severe Crohn’s disease should be treated appropriately from diagnosis with advanced therapy or surgery in select cases of limited ileal disease. Close observation may be sufficient for mild cases. Small intra-abdominal abscesses can be treated without surgery in some cases and a cooperative multi-disciplinary team working approach with radiology, surgery and dietitians is key. Anti-TNF therapy provides effective and relatively inexpensive first-line treatment but requires careful therapeutic monitoring and/or immunomodulator co-therapy to avoid immunogenicity. Those achieving remission at 1 year very often maintain this long-term. For those experiencing non-response or loss of response to anti-TNF therapy, ustekinumab or risankizumab offer appropriate second-line treatments. Biomarkers to better guide treatment decisions are urgently needed. Dietary approaches to the management of Crohn’s disease are currently evolving and may provide a future disease-modifying avenue.

## 1. Introduction

Crohn’s disease is a chronic, progressive inflammatory bowel disease characterised by segmental, transmural inflammation that can affect any part of the gastrointestinal tract, most commonly the terminal ileum and colon [1]. Symptoms may include abdominal pain, diarrhoea, rectal bleeding, nausea, fatigue, abscesses and discharge from fistulas.

The number of cases has been rising during the last decades, especially in Western Europe and North America, with an incidence rate of up to 20 new cases per 100,000 people every year [2]. Crohn’s disease presents more commonly in young adults, but it can also appear later in life. Because it often relapses and can lead to complications such as strictures, fistulae and intrabdominal abscesses, it has a significant impact on quality of life and healthcare use [3].

The previous treatment consensus highlighted in the British Society of Gastroenterology (BSG) guidelines from 2019 described three main treatment strategies for luminal Crohn’s disease [1]:

Conventional step-up, starting with steroids and immunomodulators;

Accelerated step-up, using biologics earlier in high-risk patients;

Top-down, starting directly with biologics and immunomodulators in selected cases.

These strategies aimed to achieve remission and avoid long-term bowel damage and the choice of strategy depended on several factors, such as disease location, behaviour, severity and patient preferences [1]. The concept of treat-to-target, aiming for at least mucosal healing, has, however, since gained a lot of traction and treatment paradigms appear to be shifting [4].

The current treatment options for Crohn’s disease include a wide range of advanced therapies. These include anti-TNF agents such as infliximab and adalimumab, anti-integrins like vedolizumab, IL-12/23 inhibitors like ustekinumab and interleukin (IL)-23 inhibitors such as risankizumab, mirikizumab and guselkumab. Furthermore, the small molecule JAK inhibitor upadacitinib is the only oral advanced therapy for Crohn’s disease. Treatment sequencing remains controversial and the 2024 European Crohn’s and Colitis Organisation guidelines opine that “there is currently insufficient evidence to direct how advanced therapies should be positioned in a therapeutic algorithm for luminal” Crohn’s disease [5]. Equally, the British Society of Gastroenterology 2025 guidelines do not advise on drug sequencing [6].

Although these drugs act through different mechanisms, many patients do not achieve a deep and lasting remission, with trial data often showing response in 30–40% of patients only [7]. This has led to the idea of a “ceiling of effectiveness”, meaning that even with newer therapies, there is a limit to how much benefit we can expect in real world practice [7]. Treatment intensification often does not improve outcomes once this ceiling is reached.

In this perspective, we reviewed key clinical trials from the last ten years to understand how they have shaped our thinking on treatment decisions in luminal Crohn’s disease. We chose original research studies that in our opinion have significantly influenced our decision making. In this narrative review, we purposefully did not use a systematic review approach and provide opinion rather than a full review of all available literature. We have focused on original studies as we aim to highlight how research rather than guidelines or consensus papers changed our thinking. There is no single gold standard, and many factors, such as disease type, location and patient history, must be considered when choosing the best approach. Our thoughts are based on our personal interpretation of the evidence that informs our clinical approach. Naturally, other experts may interpret the available evidence differently.

## 2. Key Studies Shaping Our Thinking

Network meta-analyses aim to compare different treatments by using direct and indirect comparisons via placebo groups [8]. These can be very helpful in the absence of head-to-head trials in providing some guidance on treatment sequencing. In a recent network meta-analysis, Barberio et al. compared the effectiveness of advanced therapies in Crohn’s disease using data from 26 clinical trials, including both induction and maintenance phases (6) [9]. This analysis separated patients into two groups: those who had never received biologic treatment before (biologic-naïve), and those who had been treated with biologics in the past.

In the biologic-naïve group, the study found that infliximab 5 mg/kg was the most effective therapy for both induction and maintenance of clinical remission. This result supports what many clinicians already see in practice: anti-TNF agents, especially infliximab, work very well in patients starting biologic therapy for the first time.

However, the authors emphasized an important limitation: infliximab was mostly studied in earlier trials that included biologic-naïve patients only, while other drugs like ustekinumab and vedolizumab were tested in more complex or refractory patients [9]. This means that the high ranking of infliximab might be partially due to the type of patients included, not just the drug’s performance. So, while infliximab remains an excellent option in early disease, the context of the trials must be considered when interpreting rankings.

In patients who had already been treated with anti-TNF therapy, the results of the network meta-analysis were different. In this group, risankizumab 600 mg was the top-ranked drug for both induction and maintenance of clinical remission [9]. This result suggests that IL-23 inhibition may be more effective than other options in patients who do not respond to or lose response to anti-TNF agents. Anti-TNF agents, which performed well in biologic-naïve patients, showed lower effectiveness in this anti-TNF experienced population. This is common in everyday clinical practice, where secondary loss of response and antibody formation often limit the long-term success of TNF inhibitors.

These results support a common strategy in Crohn’s disease: after failure of an anti-TNF agent, it may be better to switch to a treatment with a different mechanism of action. In this case, IL-23 inhibitors such as risankizumab appear to be an effective option for patients with previous biologic exposure, especially those with persistent symptoms or mucosal inflammation.

Naturally, network meta-analyses do not replace direct evidence such as head-to-head studies and results on maintenance therapy are difficult to interpret when a mixture of methodologies such as treat-through and responder re-randomisation trials are present. The network meta-analysis by Barberio et al. has given support to our strategy to start mostly with anti-TNF therapy for Crohn’s disease and often chose IL-12/23 or IL-23 biologics when anti-TNF therapy failed to deliver remission.

The SEAVUE trial was a head-to-head study comparing ustekinumab and adalimumab in patients with moderate-to-severe Crohn’s disease who were biologic-naive [10]. A total of 386 patients were randomly assigned to receive one of the two treatments, and the main goal was to assess clinical remission at week 52. The results showed that both drugs were similarly effective: 65% of patients treated with ustekinumab and 61% of those treated with adalimumab reached clinical remission. There was no statistically significant difference between the two groups. Secondary outcomes, such as endoscopic response and steroid-free remission, were also similar. Of importance is that treatment escalation was not allowed in either group, while in real world clinical practice, treatment escalation to weekly adalimumab is very common and successful when experiencing partial response [11].

Adverse events in general were equally common in both treatment groups. There were numerically fewer treatment discontinuations with ustekinumab over adalimumab (6% versus 11%).

SEAVUE was the first trial to directly compare two biologics as first-line options in luminal Crohn’s disease. Both adalimumab and ustekinumab showed a similar efficacy and safety profile. The often suggested better adverse event profile of ustekinumab was not evident in this head-to-head trial. The adalimumab biosimilar is considerably cheaper in the UK and can easily be escalated to achieve response within current funding schemes. Therefore, in our clinical opinion, an anti-TNF agent, especially adalimumab, is a sensible first-line therapy unless specific circumstances or contraindications apply. Adalimumab is currently the least expensive anti-TNF therapy option in the UK and in contrast to infliximab can be often used as monotherapy with a lower risk of immunogenicity [12]. In selected cases, ustekinumab can be used early in the disease course.

The SEQUENCE trial is a phase 3b head-to-head study comparing risankizumab and ustekinumab in patients with moderate-to-severe Crohn’s disease who had previously not responded or lost response to anti-TNF therapy [13]. Patients received standard induction and maintenance therapy with risankizumab and ustekinumab, respectively. The co-primary endpoints were clinical remission at week 24 and endoscopic response at week 48.

At week 24, more patients receiving risankizumab reached clinical remission compared to those on ustekinumab (57.1% vs. 40.6%, *p* = 0.004) [13]. At week 48, risankizumab also showed better endoscopic response (47.5% vs. 30.4%, *p* = 0.003) [13]. Both treatments had similar safety profiles. This trial provides strong evidence that IL-23 inhibition (risankizumab) may be more effective than IL-12/23 inhibition (ustekinumab) in patients who do not respond to or lose response to anti-TNF therapy. IL-23 is often upregulated in patients after anti-TNF therapy, which may explain the observed effects [14].

SEQUENCE helps with medication positioning in patients with previous anti-TNF therapy exposure. While risankizumab is clearly more effective than ustekinumab we should also consider that 40% achieved clinical remission with ustekinumab at week 24. In publicly funded healthcare systems like the UK, we need to carefully consider how limited resources are spent. Given the vast costing differences currently present between the biosimilar ustekinumab and originator risankizumab, treating every patient with previous anti-TNF therapy exposure with risankizumab may not be affordable. In our view, patients with a lesser inflammatory burden and more favourable phenotype should receive ustekinumab, while those with a high inflammatory burden or a more severe phenotype should be treated with risankizumab (Table 1 shows criteria that could be used for decision making).

When deciding on treatment sequences, clinicians should reflect that in addition to treatment efficacy, safety profiles should be considered. This is especially important for patients currently pregnant or planning pregnancy [15], those of older ages [16], those with comorbidities or frail patients [17,18], but previous histories of malignancy [19] also influence decision making.

The PROFILE trial was a randomised, open-label study that tested a personalised treatment approach in patients with newly diagnosed Crohn’s disease [20]. Researchers used a blood test biomarker (IBDhi) to divide patients into high-risk and low-risk groups, based on gene expression linked to future complications. After risk stratification, patients were randomly assigned to either top-down therapy (starting with infliximab and an immunomodulator) or step-up therapy (starting with steroids, then moving to immunomodulators and then biologics if needed). The trial did not meet its primary biomarker endpoint, there was no significant difference in the rate of steroid-free remission at 48 weeks in high-risk compared to low-risk patients. However, top-down patients had fewer hospitalisations, better symptom control, and needed fewer treatment escalations [20]. There was a striking difference in the need for surgery, which was significantly lower in the top-down group [20].

The study proved that the development of predictive biomarkers for disease severity remains challenging and prognostication in IBD remains fraught with difficulties. Any further biomarker development should be followed by rigorously controlled clinical trials to ensure their validity. While the biomarker outcome was naturally disappointing, the study clearly demonstrated that in patients with active moderate to severe Crohn’s disease, early effective treatment prevents unfavourable disease outcomes. While the study used combination infliximab and azathioprine as the main intervention, we consider it likely that other equally effective therapies will achieve similar outcomes. Of note, median time to treatment was 12 days form diagnosis. This is an achievement rarely mirrored in routine clinical practice and should be seen as aspirational target.

The LIR!C trial was a multicentre, randomised study comparing two treatment options in patients with non-complicated Crohn’s disease limited to the terminal ileum [21]. All patients were biologic-naïve and had no strictures or fistulas. They were randomised to receive either early ileocecal resection or infliximab therapy. At 12 months, 83% of patients who had surgery were in steroid- and biologic-free remission, compared to only 43% in the infliximab group [21]. The surgery group also reported good quality of life, and the procedure was generally safe and well tolerated. These results suggest that early surgery may be a strong alternative to medical therapy in well-selected patients with isolated ileal disease. Rather than reserving surgery for complications, this study shows that it can be used earlier in the disease course, potentially reducing long-term treatment burden. Long-term follow revealed decent disease control in both groups though 26% of the surgery group started TNF-therapy and 48% of the infliximab group required a resection after a median follow-up of 63 months [22].

**Figure 1:**
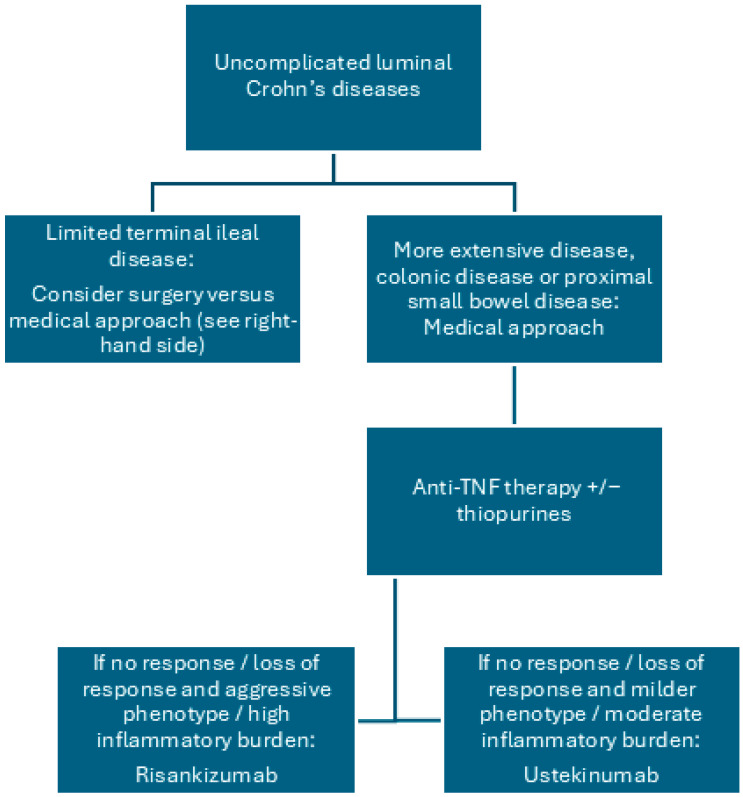
Treatment proposal for uncomplicated luminal Crohn’s disease.

Laparoscopic ileo-caecal resection is therefore an alternative to primary biologic therapy in a select cohort of patients with uncomplicated limited terminal ileal Crohn’s disease where a primary anastomosis is feasible (Figure 1).

Traditionally, intra-abdominal abscesses in Crohn’s disease have been seen as an indication for surgery. This paradigm has been challenged by prospective cohort study by Bouhnik et al., which showed that medical treatment can be effective in many of these cases [23]. The study included patients with abdominal abscesses of 20–45 mm size who were treated with a combination of antibiotics plus/minus percutaneous drainage when needed, followed by early use of anti-TNF therapy. The primary endpoint of treatment failure at week 24, defined as a need for steroids after week 12, intestinal resection, abscess recurrence or clinical relapse, was avoided in 74% [23]. After one year, over 60% of patients had avoided surgery, and their symptoms had improved [23]. This conservative approach was safe, with no increase in serious infections or complications. Patients who responded well were those with limited disease, no fistulas and good nutritional status.

These findings support the idea that surgery can be avoided in some cases of small intra-abdominal abscesses in Crohn’s disease. In selected patients, careful monitoring and a stepwise medical approach, starting with antibiotics and followed by biologics, can be effective and less invasive.

This study is a good example of how treatment decisions can be personalised, based on the location and behaviour of the disease, rather than following a fixed approach.

In contrast, milder forms of ileal Crohn’s disease may follow a benign disease course. The study by Wintjens et al. (Table 2) analysed long-term outcomes in 432 patients with Crohn’s disease, using data from the IBD South Limburg Cohort in the Netherlands [24]. All patients were followed for at least 10 years after diagnosis. By analysing the patterns of disease activity over time, the authors identified six distinct groups. One of the most important findings was that 28.2% of patients belonged to a “quiescent cluster” [24]. These patients had very few flares, did not need surgery, and did not require biologics or steroids during follow-up. Patent in the quiescent cluster had predominantly ileal disease with an inflammatory phenotype [24]. These findings are against the common belief that Crohn’s disease always becomes aggressive or needs early biologic therapy. Instead, the study suggests that a watch-and-wait approach may be appropriate for some patients, in our view, those with mild disease and limited disease extent at diagnosis. These results support the need for predictive biomarkers to help doctors understand which patients are likely to develop complications, and which one may be well without aggressive treatment. Although, after the negative results of the biomarker tested in PROFILE, this appears a long time off in the future. The Wintjens study reminds us that not all patients follow the same path, and overtreatment should be avoided in those with stable disease.

The PANTS study followed more than 1000 patients with Crohn’s disease who were starting their first anti-TNF treatment with either infliximab or adalimumab [12]. After three years, the results showed that many patients had stopped treatment, either because the drug was no longer effective or because of side effects. Only 36% of patients treated with infliximab and 28% of those on adalimumab remained on the same drug after 3 years, without needing to stop or switch. Interestingly, however, the proportion of patients in full clinical remission was largely maintained over time (infliximab 40.2% at 1 year and 34.7% at 3 years; adalimumab 35.9% at 1 year and 28.9% at 3 years), while mainly patients with a partial response discontinued treatment with TNF inhibitors [12]. One of the main reasons for treatment failure was the development of anti-drug antibodies, a process known as immunogenicity. These antibodies reduce the effect of the drug and are more common when anti-TNF therapy is used alone. The study also showed that using an immunomodulator such as azathioprine together with the anti-TNF agent helped to lower the risk of antibody development and improved treatment durability. Drug concentration at end of induction was predictive of 3-year outcomes. Those with low concentrations of the anti-TNF agent were most likely to cease TNF treatment [12]. These results highlight the limits of anti-TNF therapy over time, especially in monotherapy, and suggest that proactive therapeutic drug monitoring may help early treatment escalation and switches. They also support the use of combination therapy and early drug monitoring to maintain response in the long term.

Dietary management of Crohn’s disease is of great interest to patients, yet there are currently few evidence-based recommendations in favour of dietary management to induce or maintain remission [26]. The recently published consensus guidelines by the European Crohn’s and Colitis Organisation highlight that exclusive enteral nutrition “is effective for the induction of clinical and endoscopic remission in children and adults with mild-to-moderate” Crohn’s disease [26]. The role for maintenance therapy is currently restricted to an adjunct role. There has been growing interest in a number of exclusion diets aiming to reduce inflammatory activity. Yet, there is currently insufficient evidence to recommend any dietary approach for this purpose [26]. Bancil et al. have recently reported on the effects of a low-emulsifier diet for patients with mild to moderately active Crohn’s disease [25]. In a multi-centre, randomised, double-blind, placebo-controlled, re-supplementation trial of 154 patients with mild to moderate Crohn’s disease, all participants were blindly switched to all a low-emulsifier diet with expert dietetic input and provision of 25% of foods by the trial team [25]. The patients were then blindly assigned to re-supplementation of emulsifiers or emulsifier-free foods. The blind was well maintained and participants were not informed of the low-emulsifier aspects to prevent contamination of the intervention by participants changing their diet beyond the instructions. The primary endpoint of a 70-point reduction in the Crohn’s Disease Activity Index was achieved by 39 (49.4%) on the low-emulsifier diet versus 23 (30.7%) in the control group (*p* = 0.019) [25]. In addition, exposure to the low-emulsifier diet was associated with a higher likelihood of achieving Crohn’s Disease Activity Index remission (<150 points, adjusted risk ratio [RR] 2.1; 95% CI 1.0, 4.4; *p* = 0.042). Importantly, these findings were mirrored by an objective reduction in inflammation, as a >50% reduction in faecal calprotectin was more often achieved in patients on the low-emulsifier diet (adjusted RR 2.9; 95% CI 1.1, 8.0; *p* = 0.039) [25]. These findings show that a specific dietary approach may lead to reduced inflammation and improved symptoms. Further, larger studies with harder endpoints are required to confirm these findings. While at this moment in time, patients should not be advised to change their diet permanently to treat Crohn’s disease, with further emerging evidence this advice might need to change. A potentially very exciting avenue is the combination of biologic or small molecule therapy with an effective anti-inflammatory diet. This approach could offer additional benefits in reduction of inflammation without adding further risk on top of the risks associated with single-agent immunosuppression.

## 3. Conclusions

The treatment algorithm for Crohn’s disease evolves over time and the mentioned studies have influenced our thinking. Moderate Crohn’s disease should be treated appropriately from diagnosis with advanced therapy, or in select limited ileal disease cases, surgery. Milder cases can be safely observed, however. Small intra-abdominal abscesses can be treated without surgery in some cases. Anti-TNF therapy provides effective and relatively inexpensive first-line treatment but requires careful therapeutic monitoring and/or immunomodulator co-therapy to avoid immunogenicity. Those achieving remission at 1 year very often maintain this long-term. Those experiencing non-response or loss of response to anti-TNF therapy should be offered ustekinumab or risankizumab as second-line therapy. Biomarkers to better guide treatment decisions are urgently needed but may unfortunately not be available for several years given the current evidence. Future research should focus on identifying (bio)markers to guide treatment choice by predicting response to treatment for individual drugs. Further work should focus on examination of focused treat-to-target strategies in patients achieving clinical but not endoscopic remission. Dietary management may, in future, offer actual benefits in attempts to reduce inflammatory activity and could be a sensible combination therapy with biologics or small molecules. Naturally we have not reviewed the full breadth of the literature, and many other aspects need to be considered in individual cases. This perspective aimed to highlight a select number of important studies only.

## Figures and Tables

**Table 1:** Criteria to help chose between ustekinumab and risankizumab in patients experiencing failure of anti-TNF therapy.

	Ustekinumab Preferred	Risankizumab Preferred
Inflammatory burden as measured by C-reactive protein, albumin and/or faecal calprotectin	Moderate	High
Extent	Limited terminal ileal disease	Extensive small bowel disease
Penetrating phenotype		Preferred choice
Previous surgery	Single	Multiple
Planned pregnancy	Good safety data	Very little data

**Table 2:** Characteristics of reviewed studies.

Author/Study Title	Study Type	Participants	Main Outcome
Barberio et al. [9]	Network meta-analysis	26 clinical trials	Infliximab 5 mg/kg most effective therapy for induction and maintenance of clinical remission in treatment-naïve patients
SEAVUE [10]	Head to head trial	386 patients with moderate to severe luminal Crohn’s disease	Adalimumab and ustekinumab are equally effective in treatment-naïve patients
SEQUENCE [13]	Head to head trial	520 patients with moderate to severe luminal Crohn’s disease with previous anti-TNF therapy exposure	Risankizumab is more effective in inducing clinical and endoscopic remission than ustekinumab after exposure to anti-TNF therapy
PROFILE [20]	Open label treatment strategy trial	386 newly diagnosed patients with moderate to severe Corhn’s disease	Infliximab and thiopurine combination therapy is superior to conventional step-up therapy at 1 year
LIR!C [21,22]	Open label treatment strategy trial	143 patients with limited, uncomplicated terminal ileal Crohn’s disease	Surgical resection and infliximab treatment show similar long-term outcomes
Bouhnik et al. [23]	Open label single-arm treatment trial	190 patients with Crohn’s disease and abdominal abscesses of 20–45 mm size	60% avoided surgery after 1 year with antibiotics followed by adalimumab therapy
Wintjens et al. [24]	Observational study	432 patients with Crohn’s disease	28.2% of patients in “quiescent cluster” with very few flares, no surgery and no biologics or steroids during 10 year follow-up
PANTS [12]	Observational study	1610 patients starting anti-TNF therapy for Crohn’s disease	Combination therapy with thiopurines reduces the risk of loss of response
Bancil et al. [25]	Randomised controlled trial	154 patients with mild to moderate Crohn’s disease	A low-emulsifier diet is associated with response in Crohn’s disease symptoms and faecal calprotectin
